# Vestibular Migraine Therapy: Update and Recent Literature Review

**DOI:** 10.3390/audiolres13050064

**Published:** 2023-09-27

**Authors:** Laura Zanandrea, Bruno Colombo, Massimo Filippi

**Affiliations:** 1 Neurology Unit, IRCCS San Raffaele Scientific Institute, 20132 Milan, Italy; colombo.bruno@hsr.it (B.C.); filippi.massimo@hsr.it (M.F.); 2 Faculty of Medicine, Vita-Salute San Raffaele University, 20132 Milan, Italy

**Keywords:** vestibular migraine, vertigo, treatment, prophylaxis

## Abstract

Vestibular migraine (VM) is a neurological condition that causes vertigo and headache. It is considered the most common cause of episodic vertigo. However, specific treatments are missing, and medications currently used in VM are borrowed from migraine therapy. A comprehensive practical review of the literature assessing the evidence for abortive and preventive interventions in adults with VM was published in 2022, providing practical recommendations about VM treatment. The aim of our paper is to provide an updated overview of the current state of the art of VM treatment, illustrating new evidence available in this field. Along with traditional pharmacological preventive therapies, medications targeting the CGRP pathways have recently been investigated in terms of treatment effect in VM patients, with encouraging results. Also, there is new evidence of the efficacy of non-pharmacological interventions. However, the overall evidence base for VM treatment remains sparse.

## 1. Introduction

Vestibular migraine (VM), previously known as “migraine-associated vertigo/dizziness” or “migraine-related vestibulopathy” or “migrainous vertigo”, is a neurological condition that causes vertigo and headache. The diagnostic criteria for VM are the result of a consensus of the International Headache Society and the International Barany Society for Neurotology and are included in the appendix of the International Classification of Headache Disorders (ICHD)-3 beta version. The diagnosis requires a current or past history of migraine without aura or migraine with aura and vestibular symptoms lasting from 5 min to 72 h, with at least half of vestibular episodes associated with migrainous features; other disorders causing vestibular symptoms must be excluded [1]. VM is the most common cause of spontaneous vertigo [2,3,4]. It accounts for about 11% of patients seen in dizziness clinics and 13% of patients seen in migraine clinics [5,6]. Similar to migraine, for VM, a female preponderance is observed [7]. 

Specific treatments for VM are actually missing. Thus, preventive treatments are borrowed from migraine prevention therapy. For acute treatment, migraine analgesics are traditionally combined with antivertigo medications. Recently, a systematic review by Smyth et al. evaluated the use of pharmacological and non-pharmacological interventions for both prevention and acute treatment of VM in adult patients, with a practical, clinically oriented purpose [8]. For acute medication, the authors illustrate that triptans emerged to be less effective in treating vertigo attacks than headache attacks; only a little evidence supported the efficacy of non-invasive vagus nerve stimulation and external trigeminal nerve stimulation in reducing vertigo severity as well as headache severity. As preventive medication, Smyth et al. recommend flunarizine 10 mg daily, which proved to be the only preventive medication that demonstrated benefits for reducing headache frequency and vertigo severity compared to a control group. Among calcium channel blockers, flunarizine showed the lowest rate of adverse events, compared to cinnarizine 75 mg daily and lomerizine 10 mg daily. Also, propranolol seems to be effective in reducing headache and migraine symptoms, in contrast to metoprolol, which has a low rate of discontinuation and adverse events. The dosage of propranolol varied from 10 mg to 160 mg daily among the studies considered in the review, and no recommendation for a specific dosage is given. Tricyclics (amitriptyline 10 or 25 mg daily) should be used in patients with comorbid insomnia or comorbid depression or anxiety, as well as venlafaxine (from 37.5 up to 150 mg daily). Sodium valproate 500 mg twice daily and topiramate 25 mg twice daily or 50 mg twice daily should be used with caution, especially in women, mainly because of their adverse effects and their teratogenic effects. Lamotrigine 100 mg daily should be used only in patients who have failed multiple other options, as there is only one study supporting its efficacy in VM patients. On the contrary, acetazolamide 250 mg twice daily is not recommended as it is poorly tolerated and is associated with a high rate of discontinuation. There is a little, low certainty evidence of botulinum toxin A (155 units to 31 sites) for reducing the frequency and severity of vertigo symptoms; therefore, according to Smyth et al., botulinum toxin is likely to be more effective for headache than vestibular symptoms. Non-pharmacological therapy such as vestibular rehabilitation, including strengthening exercises, habituation exercises, canalith repositioning maneuvers, gait retraining, and sensory re-weighting tasks, should be reserved for patients with a high frequency of attacks despite optimal pharmacological therapy. Smyth et al. conclude that the overall evidence base for vestibular migraine treatment is of low quality and further research is needed.

This paper aimed at providing an updated overview of the current state of the art of VM treatment, illustrating new evidence available.

## 2. Materials and Methods

**Figure 1:**
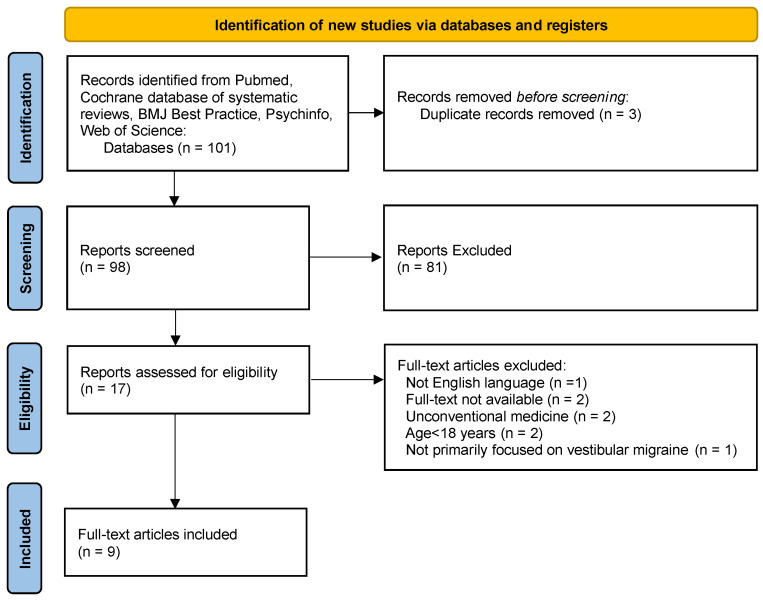
PRISMA flow diagram.

For this narrative review, we considered the most recent studies published on VM treatment. The search was performed in July 2023, and the following sources were searched: PubMed, Cochrane database of systematic reviews, BMJ Best Practice, Psychinfo and Web of Science. The following search terms were used: “Vestibular migraine” AND “treatment”, “Vestibular migraine” AND “therapy”, “Vestibular migraine” AND “prophylaxis”. We applied a filter for publication date in order to find papers published between March 2022 and July 2023. The inclusion criteria were English language and participants aged >18 years. We excluded studies on unconventional medicine or those unavailable for consultation. Our study was conducted in accordance with the Preferred Reporting Items for Systematic Reviews and Meta-Analyses (PRISMA) guidelines. Study selection is depicted in a PRISMA flow diagram (Figure 1).

## 3. Results

Articles selected for this review are shown in Table 1.

## 4. Discussion

### 4.1. Cochrane Reviews

Cochrane reviews by Webster et al. on pharmacological and non-pharmacological interventions for prophylaxis treatment and for acute treatment of VM illustrated that the overall evidence base for the treatment of this neurological condition is sparse and of low certainty [9,10,11]. Searching for evidence of the efficacy of preventive medications currently used in vestibular migraine, the authors found only three studies with a total of 209 participants that satisfied the strict inclusion criteria required for a Cochrane review: one evaluated metoprolol 95 mg daily for six months compared to placebo, and the other two evaluated flunarizine 10 mg daily for three months compared to no intervention. Because of the limited data available, meaningful conclusions were not formulated [9]. Also, evidence for acute medications for VM proved to be very sparse; only two studies were included in the review, providing evidence of low certainty about the efficacy of triptans in the acute phase of VM [10]. According to Webster et al., there is also a paucity of evidence for non-pharmacological interventions that may be used for prophylaxis of VM, including dietary interventions (probiotics), cognitive-behavioral therapy (CBT), and vestibular rehabilitation, so their role as preventives is unclear [11]. The authors conclude that further research is needed to identify interventions that can improve the symptoms of VM attacks and to determine any side effects associated with their use. 

### 4.2. Traditional Migraine Preventives

A recent prospective interventional study conducted by Islam et al. on 31 VM patients treated with flunarizine 10 mg daily for two months showed a statistically significant reduction in frequency (*p* = 0.001) and duration (*p* = 0.001) of headache as well as in frequency (*p* = 0.001), duration (*p* = 0.001), and intensity (*p* = 0.009) of vertigo. The small sample size and a missing control group make this evidence of low certainty [12]. 

A before-and-after study conducted by Oh et al. investigated the efficacy of botulinum toxin A as a preventive treatment for VM. Twenty VM patients were treated with botulinum toxin A according to the PREMPT protocol (155 units in 31 designated sites); all of the patients were on concomitant preventive medication, whose dosage was stable for at least two months prior to the study. An overall reduction in the mean monthly frequency of headache and vertigo episodes after two months after treatment was observed: the baseline mean monthly frequency of 17 migraine headaches and 14 vertigo episodes reduced to the mean monthly frequency of 9.5 (*p* = 0.001) and 8.5 (*p* < 0.001), respectively. Also, a significant reduction in Head Impact Test (HIT-6), Migraine Disability Assessment Test (MIDAS), Migraine-Specific Quality-of-Life Questionnaire (MSQ), Vertigo Severity Score (VSS), Dizziness Handicap Inventory (DHI), Beck Anxiety Inventory (BAI), and the Beck Depression Inventory (BDI) scores was observed [13]. The small sample size and the study design make the evidence of low certainty. Furthermore, the efficacy of botulinum toxin A beyond the placebo effect in VM remains unproven, as a placebo control group is missing. 

### 4.3. Medications Targeting the CGRP Pathway

In their review, Smyth et al. did not illustrate the efficacy of anti-CGRP medications in a systematic way, as they found only one study on the topic, by Hoskin et al., which did not meet the inclusion criteria. Hoskin et al. retrospectively reviewed with a qualitative approach the clinical records of follow-up visits of 25 VM patients treated with any anti-CGRP medication (fremanezumab, erenumab, galcanezumab, or ubrogepant), labeling the patient response as “significant improvement”, “moderate improvement”, “mild improvement”, or “no improvement”. They found that eight patients out of nine treated with erenumab, seven patients out of nine treated with fremanezumab, five out of five treated with galcanezumab, and one out of two treated with ubrogepant reported at least a mild improvement in vestibular symptoms [14]. This study provides low-quality evidence of the efficacy of anti-CGRP medications in VM patients because of the study design, the small sample size, the presence of concomitant preventive medication (botulinum toxin A) in some patients, and the unknown duration of treatment with an anti-CGRP medication at the time that clinical benefit was reported. 

A recent prospective observational cohort study by Russo et al. evaluated the efficacy of erenumab 140 mg, fremanezumab 225 mg, or galcanezumab 120 mg for the treatment of 50 patients with VM and previous failure or contraindication to at least three traditional migraine preventives. After 12 months of treatment, the authors observed a great reduction in vestibular symptom frequency (from 10.3 to 0.8 days, *p* < 0.001) as well as in headache frequency and headache-associated disability, with 90% of patients reporting at least a 50% reduction in vertigo frequency, 86% at least a 50% reduction in headache frequency, and 40% a MIDAS reduction of at least 50%. The clinical benefit was observed just after three months of treatment. No significant difference was found in efficacy between the three monoclonal antibodies [15]. The small sample size and the observational design without a control group make the evidence of low certainty. 

### 4.4. Non-Pharmacological Treatments

Sun et al. explored the treatment outcome of resistance exercise on symptoms of VM, in particular vertigo and dizziness, in a randomized clinical trial. Resistance exercise is a clinical intervention involving physical exercise that induces muscle contraction by using an external resistant force in order to increase bone density, strength, and muscular mass [16]. A total of 312 VM patients were randomized into either resistance exercise or relaxation control groups, receiving respective interventions for 60 minutes two times per week for 12 weeks. Patients in the relaxation control group were instructed to perform gradual muscle relaxation without muscle strengthening or aerobic exercise. Eleven participants in the resistance exercise group and 15 in the relaxation control group were lost at follow-up and, therefore, were not included in the analysis. The authors found that resistance exercise was slightly more effective for alleviating VM symptoms, according to DHI scores, than relaxation control after two months of treatment (*p* = 0.02). After four months of treatment, not only was the DHI score reduction greater in the resistance exercise group than in the relaxation control group (*p* = 0.01), but also the reduction in VSS scores (*p* = 0.03), BDI scores (*p* = 0.04), and BAI scores (*p* = 0.03), as well as in the number of vertiginous attacks in the previous week (−1.8 vs −1.1, *p* = 0.02) [17]. However, the evidence is of low quality, as it is not clear whether patients were taking any concomitant preventive medication.

### 4.5. Pregnancy and Breastfeeding

A recent review by Teelucksingh et al. explored existing evidence-based treatment options for VM during pregnancy and breastfeeding. Paracetamol, non-steroidal anti-inflammatory drugs up to 30–32 weeks of gestation, and sumatriptan are described as safe acute medications during pregnancy. Propranolol and amitriptyline are reported as first-line preventive medications with a robust safety profile in pregnancy and breastfeeding. Because of adverse events, acetazolamide is reported as a second-line preventive medication with adequate safety data, particularly after the first trimester of pregnancy. The occipital nerve block might be a valid option for pregnant or breastfeeding women, but evidence of efficacy is lacking. On the contrary, among traditional medications, according to Teelucksingh et al., calcium channel blockers and botulinum toxin A are not recommended in pregnancy and breastfeeding because of insufficient safety data, while high-dose aspirin, opioids, ergotamine derivates, angiotensin-converting enzyme (ACE) inhibitors, angiotensin receptor blockers, and anticonvulsants must not be used in these patients as they are unsafe. Among emerging therapies, magnesium is safe, although no evidence about its efficacy in VM is reported, while safety data are lacking for anti-CGRP medications and cyproheptadine. Safety data as well as efficacy data for neuromodulation devices are missing. Among non-pharmacological preventive interventions, acupuncture is considered safe in pregnancy [18]. 

## 5. Conclusions

Despite the new evidence available, the overall evidence base for the treatment of VM remains sparse. The efficacy of flunarizine and botulinum toxin A as preventive medications in VM patients has recently been investigated, with encouraging results. Also, medications targeting the CGRP pathway have recently shown clinical benefits in VM patients. In the field of non-pharmacological interventions, resistance exercise has recently been described as effective for reducing vestibular symptoms. However, because of several limitations of the studies, the evidence remains of low quality. Therefore, this paper highlights the need for further studies in order to identify effective treatments for VM patients.

## Figures and Tables

**Table 1:** Studies considered for this review.

First Author	Study Design	Patients (n)	Treatment Investigated
Webster	Cochrane review	209	Pharmacological preventive interventions
Webster	Cochrane review	133	Pharmacological acute interventions
Webster	Cochrane review	319	Non-pharmacological interventions
Islam	Prospective interventional	31	Flunarizine
Oh	Prospective interventional	20	Botulinum toxin A
Hoskin	Retrospective chart review	25	Anti-CGRP medications
Russo	Prospective observational	50	Anti-CGRP antibodies
Sun	Randomized clinical trial	315	Resistance exercise
Teelucksingh	Narrative review	-	Pharmacological and non-pharmacological interventions

## Data Availability

Not applicable.
